# Characterization of the emerging zoonotic pathogen *Arcobacter thereius* by whole genome sequencing and comparative genomics

**DOI:** 10.1371/journal.pone.0180493

**Published:** 2017-07-03

**Authors:** Francesca Rovetto, Aurélien Carlier, Anne-Marie Van den Abeele, Koen Illeghems, Filip Van Nieuwerburgh, Luca Cocolin, Kurt Houf

**Affiliations:** 1 Department of Veterinary Public Health and Food Safety, Faculty of Veterinary Medicine, Ghent University, Salisburylaan 133, Merelbeke, Belgium; 2 Department of Forestry, Agriculture and Food Sciences, University of Torino, Largo Braccini 2, Grugliasco, Italy; 3 Laboratory of Microbiology, Faculty of Sciences, Ghent University, K. L. Ledeganckstraat 35, Ghent, Belgium; 4 Microbiology laboratory, Saint-Lucas Hospital, Groenebriel 1, Ghent, Belgium; 5 Laboratory of Pharmaceutical Biotechnology, Faculty of Pharmaceutical Sciences, Ghent University, Harelbekestraat 72, Ghent, Belgium; Massey University, NEW ZEALAND

## Abstract

Four *Arcobacter* species have been associated with human disease, and based on current knowledge, these Gram negative bacteria are considered as potential food and waterborne zoonotic pathogens. At present, only the genome of the species *Arcobacter butzleri* has been analysed, and still little is known about their physiology and genetics. The species *Arcobacter thereius* has first been isolated from tissue of aborted piglets, duck and pig faeces, and recently from stool of human patients with enteritis. In the present study, the complete genome and analysis of the *A*. *thereius* type strain LMG24486^T^, as well as the comparative genome analysis with 8 other *A*. *thereius* strains are presented. Genome analysis revealed metabolic pathways for the utilization of amino acids, which represent the main source of energy, together with the presence of genes encoding for respiration-associated and chemotaxis proteins. Comparative genome analysis with the *A*. *butzleri* type strain RM4018 revealed a large correlation, though also unique features. Furthermore, *in silico* DDH and ANI based analysis of the nine *A*. *thereius* strains disclosed clustering into two closely related genotypes. No discriminatory differences in genome content nor phenotypic behaviour were detected, though recently the species *Arcobacter porcinus* was proposed to encompass part of the formerly identified *Arcobacter thereius* strains. The report of the presence of virulence associated genes in *A*. *thereius*, the presence of antibiotic resistance genes, verified by *in vitro* susceptibility testing, as well as other pathogenic related relevant features, support the classification of *A*. *thereius* as an emerging pathogen.

## Introduction

The genus *Arcobacter* was created in 1991 as a second genus within the family Campylobacteraceae to include bacteria which differ from the closely related *Campylobacter* species by their aerotolerance and ability to grow at temperatures below 30° [1]. At the time of writing, 21 species have been characterized including the new species *Arcobacter ebronensis*, *Arcobacter aquimarinus*, *Arcobacter faecis*, *Arcobacter lanthierii* and *Arcobacter pacificus* [2–5]. These species are isolated from environmental matrices or shellfish. Six species are commonly isolated from food of animal sources across the world [6]. In particular the species *Arcobacter butzleri*, *Arcobacter cryaerophilus* and *Arcobacter skirrowii* are incriminated as food and waterborne pathogens for humans [7]. In contrast to animals where infection is asymptomatic, *Arcobacter* infection in humans seems to cause enteritis and sometimes bacteraemia, with clinical signs similar to those of campylobacteriosis, but with a higher frequency of persistent watery diarrhoea [8–10]. Contaminated drinking water and the manipulation or consumption of raw or undercooked food are likely to be the infection sources [7].

Recently, the species *Arcobacter thereius* was isolated from the stool of two hospitalized patients with symptoms of enteritis [9]. This species had first been isolated during a Danish study on the prevalence of campylobacteria in ducks and in internal organs of aborted piglets [11,12]. Some of the isolated Gram negative, rod-shaped, slightly curved, not able to degrade urea, non-spore-forming, oxidase, catalase and nitrate reduction positive, bacteria clustered in a distinct phenon within the genus *Arcobacter*, and were later characterized by Houf et.al. [13] as representing a novel species, *A*. *thereius*. Though the species has been isolated relatively frequently from the faeces of healthy pigs, still almost nothing is known about its behaviour [14]. Houf et al. already reported its lack of growth at 37°C under conditions which cultivate other host-associated arcobacters [13], and the generally slow and difficult *in vitro* culture has troubled many researchers since. Furthermore, in contrast to the other mammal-associated species, *A*. *thereius* isolates are hard to type at strain level, in fact pulsed-field gel electrophoresis (PFGE), amplified fragment-length polymorphism (AFLP) and enterobacterial repetitive intergenic consensus PCR (ERIC-PCR) showed to be not discriminatory for *A*. *thereius* [15], and the already identified virulence associated genes in the other human associated *Arcobacter* species seem to be lacking in *A*. *thereius* [16]. Since *A*. *thereius* is *in vitro* non-fermenting, nor oxidising carbohydrates, as for the other members of the family Campylobacteraceae, genome analysis represents an ultimate approach to elucidate this species full potential.

The present study presents the full genome analysis of the *A*. *thereius* type strain LMG24486^T^, as well as the sequences of eight other strains originating from different matrices. The genome of the type strain is analysed with a focus on metabolic pathways, virulence factors, antibiotic resistance genes and adaptation to the environment. The genome is then compared with the only other yet available full genome of a human associated species: *A*. *butzleri* type strain RM4018 (= LMG10828^T^) [17]. Subsequently, strain variation in *A*. *thereius* is assessed in order to comprehend the previously reported strain high heterogeneity [13].

## Materials and methods

### 1. Bacterial strains and growth conditions

For genome analysis, nine *A*. *thereius* strains were included (Table 1). Five strains were isolated during a Danish surveillance study: strains DU19 and DU22 from duck faeces, and strains LMG24487, 11743–4 and LMG24486^T^, including the type strain, from the internal organs of spontaneous porcine abortions [11,12]. Four unrelated strains, isolated from porcine faeces, were randomly chosen from the *Arcobacter* strain bank of the Department of Veterinary Public Health and Food Safety, Faculty of Veterinary, Ghent University, Belgium [14]. Both *A*. *thereius* strains previously isolated from humans, have been preserved on pearls, and showed to be no longer culturable, and therefore were not included in the present study.

**Table 1:** General features of the *Arcobacter thereius* genome sequences studied.

	Origin	Total bases (bp)	Number of scaffolds	G/C content (%)	Average scaffold size (bp)	N50
*A*. *thereius* DU19	cloacal content duck	1,883,443	21	26.99	89,687	263,408
*A*. *thereius* LMG24487	piglet, aborted foetus	2,142,650	62	26.98	34,582	123,374
*A*. *thereius* 11743–4	piglet, aborted foetus	1,967,717	10	27.10	197,275	1,193,917
*A*. *thereius* 440	pig faeces	1,929,158	10	26.95	193,814	507,023
*A*. *thereius* 213	pig faeces	1,781,662	11	27.10	161,969	216,430
*A*. *thereius* 452	pig faeces	1,974,041	17	26.72	116,120	391,233
*A*. *thereius* 216	pig faeces	1,778,866	12	27.09	148,239	302,501
*A*. *thereius* LMG24486^T^	piglet, aborted foetus	1,903,306	1	26.93	1,909,684	1,909,684
*A*. *thereius* DU22	cloacal content duck	2,012,225	17	26.78	118,366	493,271

Strains were stored at -80°C in full-horse blood. Re-cultivation was performed by inoculation of 10 μL from the stock onto blood agar plates, and incubation at 28°C for 72 h in microaerobic conditions by evacuating 80% of the normal atmosphere and introducing a gas mixture of 8% CO_2_, 8% H_2_ and 84% N_2_ into a jar.

### 2. Genome analysis

#### 2.1. DNA extraction and genome sequencing

DNA extraction was performed using the DNeasy Tissue Kit (50) (Qiagen, Venlo, The Netherlands) according to the manufacturer’s instructions, followed by evaluation of the DNA integrity by size separation of 10 μL of each DNA sample by agarose gel electrophoresis (1.5%). An Illumina paired-end library was generated from 1 μg of genomic DNA for each of the nine strains. The DNA was fragmented to 800 bp using Covaris S2 sonication (Covaris, MA, USA) and a sequence library was made for each sample using the NEBNext Ultra DNA Library Prep Kit (New England Biolabs, Ipswich, MA, USA). During the library preparation, a size selection was performed before and after the enrichment PCR using an Invitrogen 1% E-gel (ThermoFisher Scientific, Waltham, MA, USA) to select for fragments between 850 and 1200 bp. The libraries were equimolarly pooled and were sequenced on an Illumina MiSeq sequencer (Illumina, San Diego, CA, USA), generating 2x300 bp paired-end reads for each sequenced fragment. Base calling and primary quality assessments were performed using Illumina’s Basespace genomics cloud computing environment. Using CLC bio Genomics Workbench version 8.0, the reads were trimmed based on quality scores, library prep adapters were removed, and resulting sequences shorter than 100 nt were discarded. The quality trimmed reads were used to perform a *de novo* assembly with CLC bio Genomics Workbench version 8.0 using standard settings. The resulting scaffolds were further extended and joined into a final set by SSPACE version 2.0 [18] using all quality filtered reads. The remaining gaps inside the final scaffolds were partially or completely removed using GapFiller version 1.10 [19] and the quality filtered reads.

Additionally, the genome of the type strain, *A*. *thereius* LMG24486^T^ was sequenced using PacBio technology [20], following the chemistry Polymerase P6/ Chemistry C4.0 and the type of the library “20 kb Template Preparation Using BluePippin Size-Selection system” protocol from PacBio (ref 100-286-000-07) with the actual fragment distribution on BioAnalyser >12kb. The long reads, together with the scaffolds generated by CLC bio Genomics Workbench starting from Illumina paired-end data, were submitted to a hybrid assembly using SSpace-Longread version 1.1 [21]. The gaps in the final scaffolds were partially or completely closed using GapFiller.

#### 2.2. Genome annotation and analysis

The assembled genome sequences of the nine *A*. *thereius* strains were annotated using a local installation of the Prokka platform v1.11 [22], using the feature prediction tools Prodigal v2.60 [23], ARAGORN v1.2 [24], Barrnap v.0.5 (http://www.vicbioinformatics.com/software.barrnap.shtml), Infernal v1.1.1, and SignalP v4.1 [25,26]. Further, protein-encoding sequences (CDS) were annotated by BLAST analysis (e-value cut-off of 10^−6^ and 50% of identity) using all available bacterial proteins in the UniProt database 2015_04 [27] and HMMER analysis using the Pfam [28] and TIGRFAM [29] databases. The genome sequences of the *A*. *thereius* strains were also annotated by the Rapid Annotation using Subsystem Technology (RAST) [30,31] platform. Annotations from Prokka and RAST were merged in one final annotation file in order to reduce the number of hypothetical proteins. CDS of interest were additionally analysed by PSI-BLAST (default setting) and metabolic pathways were constructed using the Kyoto Encyclopedia of Genes and Genomes (KEGG) database [32]. Plasmid sequences were identified using PlasmidFinder version 1.2 [33] and the PATRIC [34] plasmid database (plasmid_seq, version 30/6/2014) and prophage regions were detected using PHAST [35]. Antibiotic resistance genes were searched for by BLASTn analysis using the antibiotic resistance gene-annotation database (ARG-ANNOT; [36]) using default parameters and the comprehensive antibiotic resistance database (CARD, [37]) by BLASTp analysis with an default e-value cut-off of 10^−30^; virulence factors were identified by BLASTn analysis using the human pathogenic bacteria virulence factor database (VFDB;[38–40]) with default parameters.

For *A*. *thereius* LMG 24486^T^, clustered regularly insterspaced short palindromic repeat (CRISPR) regions were identified through CRISPRFinder [41] and a genome plot was generated with DNAPlotter [42]. The origin of chromosomal replication was predicted with the Ori-Finder tool [43]. Genomic islands were searched for with IslandViewer [44] and bacteriocins with BAGEL3 [45].

#### 2.3 Comparative genome analysis

A maximum likelihood phylogenetic tree of a concatenated alignment of 239 single-copy orthologs was reconstructed. One-to-one ortholog genes were obtained by comparing a genome database comprising the *A*. *thereius* strains that have been sequenced in this study, all other *Arcobacter* genomes available from NCBI and *Campylobacter jejuni* NCTC11168 as outgroup. Orthologues were predicted using the OrthoMCL software v1.4 [46] using the following settings: BLASTp e-value cut-off: 10^−6^; identity cut-off 50%; reciprocal hit length cut-off: 50%. Further, in order to include only orthologs without evidence of recombination or gene conversion, a stringent filtering step was added. Briefly, the probability of recombination for each orthologous group was calculated using the PhiPack software [47], and discarding alignments without sufficient phylogenetic signal or with a p-value < 0.05 (with 1000 permutations). Nucleotide corresponding to 239 single copy ortholog sequence sets were aligned with MUSCLE [48], trimmed with TrimAL [49] to remove columns with >80% gaps and concatenated. The maximum likelihood tree was built with RaxML [50] using the GTRGAMMA substitution model and 100 bootstrap replicates.

Comparative analysis between *A*. *thereius* LMG24486^T^ and *A*. *butzleri* RM4018 and within the *A*. *thereius* strains were performed using the OrthoMCL software v1.4 [46] using the following settings: BLASTp e-value cut-off: 10^−6^; identity cut-off 50%; reciprocal hit length cut-off: 50%. EDGAR [51] comparative genome analysis was also used to obtain the core and the pan genome plot of *A*. *thereius* species and to construct a synteny plot between *A*. *thereius* LMG24486^T^ and *A*. *butzleri* RM4018 following default parameters.

A phylogenetic analysis using MEGA 6 [52] was performed based on the amino acid sequence of the virulence genes that were in common in all nine *A*. *thereius* strains. Further, amino acid sequences of *Campylobacter* and/or *Helicobacter* species were included in the analysis in the cases where an appropriate amino acid sequence could be retrieved from the NCBI data repository. Each dendrogram was made using the Neighbor-Joining method. Bootstrap values of >50% generated from 100 replicates are shown next to the branches. The evolutionary distance were computed using the p-distance method and are in the units of the number of amino acid differences per site.

The Average Nucleotide Identity based on BLAST+ calculation (ANIb) was performed for the nine *A*. *thereius* strains using JSpecies Web Server (JSpace WS) with default parameters [53]. The genome of *A*. *butzleri* RM4018 and *A*. *cibarius* LMG21996 were used as an outgroup. In addition, *in silico* DNA-DNA hybridisation was calculated for all *A*. *thereius* strains using the genome-to-genome distance calculator following as alignment method the recommended GGDC 2 BLAST+ [54]. Genomes of *A*. *butzleri* RM4018 and *A*. *cibarius* LMG21996 were included as comparison.

MLST profiles were obtained for the nine *A*. *thereius* strains using the *Arcobacter* pubMLST database (http://pubmlst.org/Arcobacter/) and according to the protocol of Miller et al. [55]. The nucleotides sequences were aligned and concatenated in the order *aspA*, *atpA*, *glnA*, *gltA*, *pmg* and *tkt*. The gene *glyA* was however not included as it was absent in *A*. *thereius* strains 213, 216, 440, 452 and 11743–4. A dendrogram was constructed with MEGA 6 using the Neighbor-joining method. Bootstrap values of >50% generated from 100 replicates are shown next to the branches. The evolutionary distance were computed using the p-distance method and are in units of the number of bases differences per site.

Alignment of the nine genomes of *A*. *thereius* has been generate with ProgressiveMauve [56] using default parameters. Clusters of orthologous group families (COG) were assigned, for all the nine *A*. *thereius* strains, using the rps-BLAST program against the NCBI Conserved Domain Database (CDD) and with an e-value cut-off of 1.0 × 10^−3^. Only the top hits were retained and the genes belonging to each COG categories were counted, and the distribution were compared using a Pearson chi-squared test using the IBM SPSS statistic program v23.

#### 2.4 Data availability

The annotated genome sequences were deposited in the DDBJ/EMBL/GenBank database. The sequencing projects and accession numbers are listed in Table 2.

**Table 2:** Genbank accession numbers of *A*. *thereius* genomes.

	Locus tag	Bioproject ID	Accession number
A. *thereius* DU19	AAX30	PRJNA283166	LCUI00000000
A. *thereius* LMG24487	AAX27	PRJNA283164	LCUH00000000
A. *thereius* 11743–4	AAX28	PRJNA283165	LDIR00000000
A. *thereius* 440	AAX25	PRJNA283162	LDIS00000000
A. *thereius* 213	AAW29	PRJNA282911	LCSK00000000
A. *thereius* 452	AAX26	PRJNA283161	LCUK00000000
A. *thereius* 216	AAW30	PRJNA282917	LCSL00000000
A. *thereius* LMG24486^T^	AA347	PRJNA283692	LLKQ00000000
A. *thereius* DU22	AAX29	PRJNA283167	LCUJ00000000

### 3. Growth, motility and antimicrobial susceptibility of *Arcobacter thereius*.

The nine *A*. *thereius* strains included in the present study were tested for antimicrobial susceptibility, growth and motility capacity. The latter two parameters were tested under different temperature and atmospheric conditions as shown in Table 3. Briefly, cell suspensions of 1 McFarland for each strains were prepared in 2 mL Peptone Water (Oxoid, Basingstoke, UK). For the growth test, a drop of 30 μL of each suspension was placed on blood agar plates. For the motility test, a 100 μl drop of each suspension was placed in the centre of a Petri dish containing a semi-solid media (24 g/L *Arcobacter* broth, 3 g/L Agar Technical n° 3 (Oxoid)).

**Table 3:** Characteristics of the phenotypic behaviour and distribution of antimicrobial susceptibility of the nine *A*. *thereius* strains sequenced in this study. Susceptibility breakpoint (mg/L): erythromycin: ≤ 8; ciprofloxacin: ≤ 0,5; ampicillin: ≤ 2; tetracycline: ≤ 2; gentamicin: ≤ 2; streptomycin: ≤ 16; chloramphenicol: ≤ 8; spectinomycin: ≤ 32.

Characteristic	*A*. *thereius* LMG24486^T^	*A*. *thereiu*s LMG24487	*A*. *thereius* 11743–4	*A*. *thereius* DU19	*A*. *thereius* DU22DU22	*A*. *thereius* 213213	*A*. *thereius* 216216	*A*. *thereius* 452452	*A*. *thereius* 440440
**Growth Test**									
Growth at 28° C A*	+	+	+	+	+	+	+	+	+
Growth at 28°C MA**	+	+	+	+	+	+	+	+	+
Growth at 28°C AN***	-	-	-	-	+	-	+	-	+
Growth at 37°C A	-	-	-	-	-	-	-	-	-
Growth at 37°C MA	-	-	-	-	-	-	-	-	-
Growth at 37°C AN	-	-	-	-	-	-	-	-	-
Growth at 42°C A	-	-	-	-	-	-	-	-	-
Growth at 42°C MA	-	-	-	-	-	-	-	-	-
Growth at 42°C AN	-	-	-	-	-	-	-	-	-
**Motility test**									
Motility at 28°C A	+	+	+	+	+	+	+	+	+
Motility at 28°C MA	+	+	+	+	+	+	+	+	+
Motility at 28°C AN	-	-	-	-	-	-	-	-	-
Motility at 37°C A	-	-	-	-	-	-	-	-	-
Motility at 37°C MA	-	-	-	-	-	-	-	-	-
Motility at 37°C AN	-	-	-	-	-	-	-	-	-
**Antimicrobial Susceptibility**									
Erythromycin (48h)	2	1	4	1	1	1,5	1,5	2	2
Ciprofloxacin (48h)	0,25	0,25	>32	0,25	0,12	0,12	0,5	0,25	0,25
Ampicillin (48h)	4	2	6	1,5	1,5	1	2	4	6
Tetracycline (48h)	0,5	0,38	6	0,5	0,8	0,19	0,38	0,5	4
Gentamicin (48h)	1	1	0,75	2	0,5	0,5	1	1	1
Streptomycin (48h)	8	192	48	12	12	4	6	8	8
Chloramphenicol (48h)	8	6	12	3	3	4	6	4	6
Spectinomycin (48h)	>1024	>1024	>1024	>1024	>1024	>1024	>1024	24	>1024

*A: aerobic condition

**MA: microaerobic condition

***AN: anaerobic condition

Based on the genomic findings, antimicrobial susceptibility to eight antibiotics: erythromycin, ciprofloxacin, ampicillin, tetracycline, gentamicin, streptomycin, chloramphenicol and spectinomycin was determined by the gradient strip diffusion method as previously described by Van den Abeele et al. [57]. In brief, an inoculum of 1 McFarland in 0.9% NaCl was prepared, plated on Mueller Hinton agar supplemented with horse blood (MH II-F agar, bioMérieux, Marcy L’étoile, France), and incubated at 35°C in microaerobic atmosphere for 48 hours. Quality control strains *Campylobacter jejuni* ATCC33560 and *A*. *butzleri* RM4018 were included. Due to the fastidious growth, plates were read after 48 hours. EUCAST breakpoints were used for erythromycin, ciprofloxacin and tetracycline (*Campylobacter coli*), ampicillin, (non-species-related PK/PD), gentamicin and chloramphenicol (*Enterobacteriaceae*) and spectinomycin (*Neisseria gonorrhoeae*) [58]. For streptomycin, NARMS resistance breakpoints were applied [59].

## Results and discussion

### 1. General genomic features of the type strain, *Arcobacter thereius* LMG 24486^T^

#### 1.1. Genome assembly and annotation

**Fig 1:**
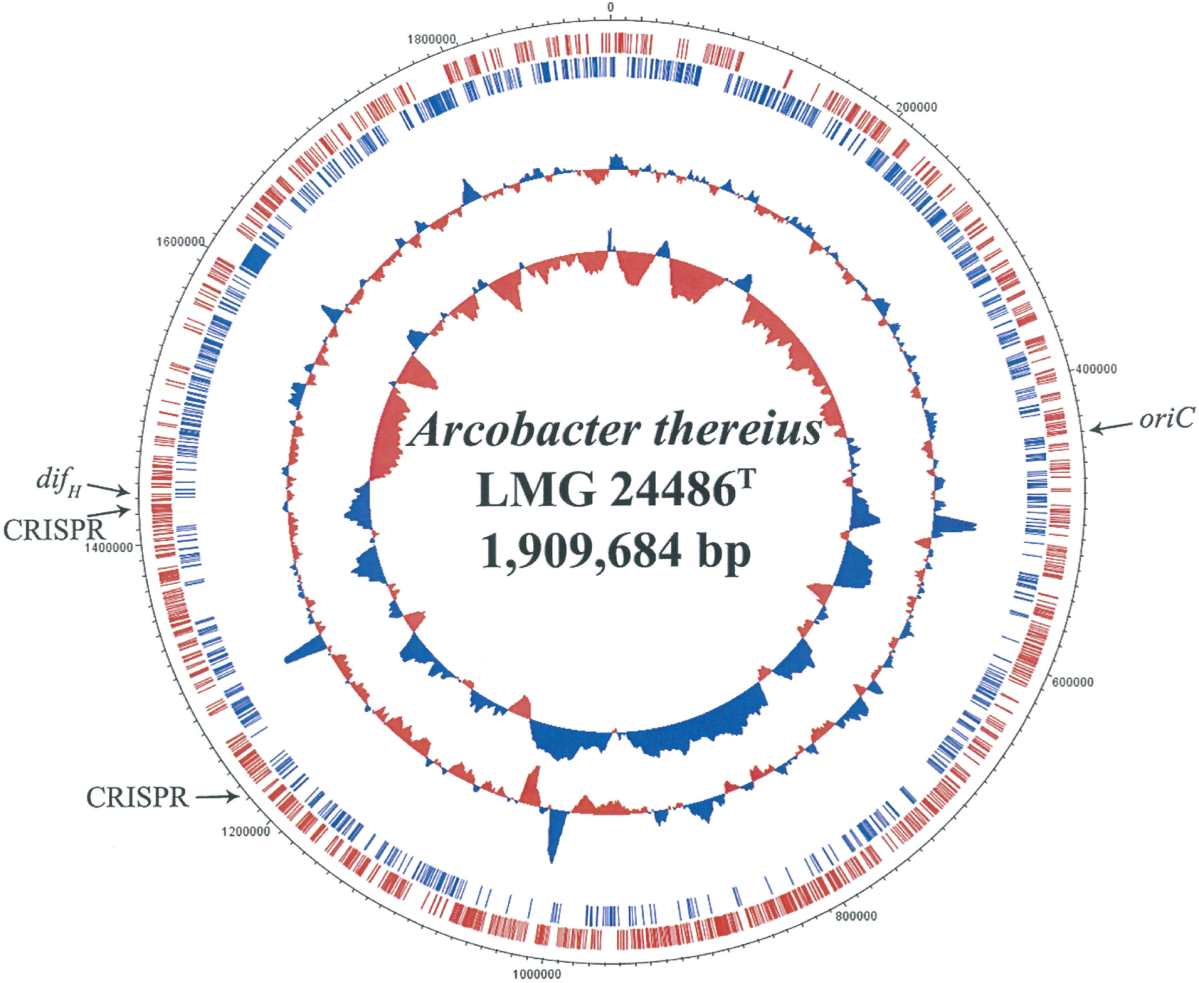
General features of the *Arcobacter thereius* LMG24486^T^ genome sequence. The circles represent (from the outside to the inside): circle 1, position (bp); circle 2, CDS transcribed clockwise; circle 3, CDS transcribed anti-clockwise; circle 4, GC content plotted using default settings; circle 5, GC skew plotted using a window size of 25,000 and default step size. The predicted *oriC*, *dif*_*H*_ site, and CRISPR sequences are indicated by black arrows. The *difH* site had a consecutive 31-bp sequence match with that of *A*. *butzleri* RM4018 [61].

Hybrid sequence assembly of the Illumina paired-end reads and PacBio long reads resulted in a single contig representing the circular chromosome of *A*. *thereius* LMG24486^T^, for a total sequence length of 1,909,306 bp with a GC content of 26.93% (Tables 1 and 4, Fig 1). The absence of plasmids in *A*. *thereius* has been already previously reported, and is confirmed in the present study [60]. Genome annotation revealed the presence of 1925 protein coding genes (CDS), three rRNA operons (all 16S rRNA genes were identical), and 46 tRNA genes. Three Cas/CRISPR regions were found in the genome of *A*. *thereius* LMG24486^T^, consisting of 28 (position 1,211,914–1,213,792), 4 (position 1,242,675–1,243,017), and 27 (position 1,243,120–1,245,145) spacer sequences, respectively (Fig 1; S1 Table). The genome sequence of *A*. *thereius* LMG24486^T^ contained no predicted bacteriocin-associated genes, prophages, and no genomic islands.

**Table 4:** Genome architecture of *A*. *thereius* LMG24486^T^.

	*A*. *thereius* LMG24486^T^
Genome size (bp)	1,909,306
GC content (%)	26.93
Number of plasmids	0
Number of CDS	1925
Coding density (%)	93.00
Average gene length (bp)	922
Number of rRNA operons	3 x 16S-23S-5S
Number of tRNAs	46
Number of tmRNAs	1
Number of transposases	4

### 2. Central metabolism

#### 2.1. Sugar metabolism

*Arcobacter thereius* LMG24486^T^ is unable to metabolize carbohydrates through the Embden Meyerhof-Parnas pathway due to the absence of genes encoding for the key enzymes 6-phosphofructokinase and glucose-1-phosphate phosphodismutase. However, as all genes encoding for the other enzymes necessary in glycolysis and a gene encoding fructose-1,6-bisphosphatase are present (AA347_01347; AA347_01345; AA347_00800; AA347_01516; AA347_00648; AA347_00647; AA347_00887; AA347_00564; AA347_00705), this suggests that the Embdem Meyerhof pathway operates towards gluconeogenesis. This functionality has also been described in the genome of the asaccharolytic *Campylobacter jejuni* [62]. All genes encoding enzymes of the non-oxidative branch of the pentose phosphate pathway (PPP) were present (AA347_01712; AA347_00012; AA347_01343; AA347_00280; AA347_00243; AA347_01540), indicating that sugars can be metabolized using this pathway, while the oxidative branch of the PPP is not active. Other pathways that allow bacteria to metabolize sugars, such as the Entner-Doudoroff pathway are not present. In *A*. *thereius*, a complete pathway for the degradation of pyruvate, which can be formed through amino acid degradation, is present (see below). A gene cluster encoding the pyruvate dehydrogenase complex allowing formation of acetyl-CoA is present (AA347_01751, AA347_01752, and AA347_01753). The latter can be channelled into the tricarboxylic acid (TCA) cycle or it can be dehydrogenated to acetate by the aldehyde dehydrogenase (AA347_00799). Further, oxaloacetate can be formed from pyruvate by carboxylation by pyruvate carboxylase (AA347_01516). Oxaloacetate can be channelled in the TCA cycle if the quantity of energy is low or it can be converted to glucose if the energy charge is high [63].

All genes encoding the enzymes of the TCA were detected, except for the one encoding the succinyl-CoA synthetase. This conformation has also been reported previously in *A*. *butzleri* RM4018 [17] and other species of the family Epsilonproteobacteria, such as *Helicobacter pylori* [64], *Campylobacter coli* RM2228, *C*. *upsaliensis* RM3195 and *C*. *lari* RM2100 [65]. In contrast, the genome sequence of *C*. *jejuni* RM1221 contains all genes encoding the TCA [64,65].

#### 2.2. Amino acid metabolism

Since *A*. *thereius* LMG24486^T^ is unable to use carbohydrates as an energy source, alternative metabolic pathways are needed. Amino acids and their degradation products seems to be good candidates since they are present in the habitat of arcobacters, including the animal and human gut. The genome sequence of *A*. *thereius* LMG24486^T^ harbours genes encoding different amino acid transporters, such as symporters specific for glutamate and aspartate/sodium (AA347_00247), an arginine/ornithine antiporter (AA347_00156; AA347_00225; AA347_00537; AA347_01038), and a methionine ABC transporter (AA347_00336). This methionine transporter is absent in *A*. *butzleri* RM4018 genome, and it could be specific for *A*. *thereius*. Furthermore, nine genes were identified encoding several ABC transporter ATP-binding proteins (AA347_00335; AA347_00462; AA347_00463; AA347_01165; AA347_01313; AA347_01549; AA347_01654; AA347_01671) that could not be further specified based on genome annotation. Nineteen transporters that are present in the genome of *A*. *butzleri* RM4018 were absent in all nine *A*. *thereius* strains sequenced. These comprised 17 ABC transporter ATP-binding proteins, one sodium:alanine symporter and a sodium:hydrogen antiporter. These additional transporters might allow *A*. *butzleri* to use more extracellular substrates compared to *A*. *thereius*. For *A*. *thereius* LMG24486^T^, four genes encoding aminopeptidases were retrieved, encompassing a leucine aminopeptidase (*pepA;* AA347_01678), a methionine aminopeptidase (*map*, AA347_01831), one generic aminopeptidase (AA347_00091) and an oligopeptidase A (*prlC*, AA347_01499) with a broad specificity.

**Fig 2:**
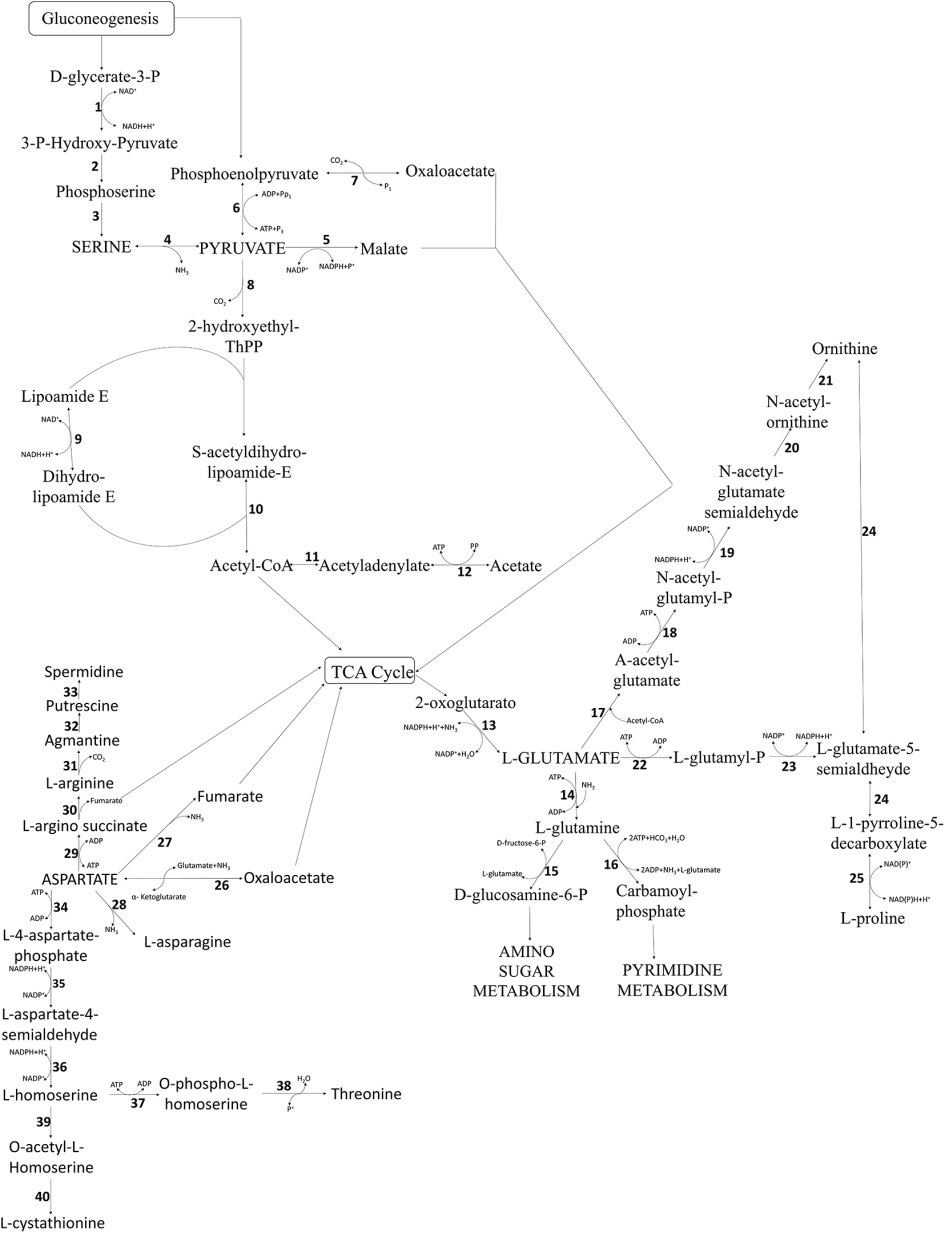
The proteolytic metabolism of *Arcobacter thereius* LMG24486^T^. **(1)** D-3-phosphoglycerate dehydrogenase (AA347_00201); **(2)** Phosphoserine aminotransferase (AA347_00366); **(3)** Phosphoserine phosphatase (AA347_00244); **(4)** L-serine deaminase (AA347_00179); **(5)** Malate dehydrogenase (AA347_01362); **(6)** Pyruvate kinase (AA347_00705); **(7)** Phosphoenolpyruvate carboxykinase (AA347_01523); **(8)** Pyruvate dehydrogenase E1 component (AA347_01757); **(9)** Dihydrolipoyl dehydrogenase (AA347_01759); **(10)** Dihydrolipoyllysine-residue acetyltransferase (AA347_01758); **(11)** Acetyl-coenzyme A synthetase (AA347_00006); **(12)** Aldehyde dehydrogenase (AA347_00801); **(13)** NADP-specific glutamate dehydrogenase (AA347_00755); **(14)** Glutamine synthetase 1 (AA347_01959); **(15)** glucosamine 6-phosphate synthase (AA347_00043); **(16)** Carbamoyl-phosphate synthase small chain, Carbamoyl-phosphate synthase arginine-specific large chain (AA347_00362; AA347_01184); **(17)** Amino-acid acetyltransferase (AA347_01854); **(18)** Acetylglutamate kinase (AA347_00182); **(19)** N-acetyl-gamma-glutamyl-phosphate reductase (AA347_01878); **(20)** Acetylornithine aminotransferase (AA347_00770); **(21)** Arginine biosynthesis (AA347_01036); **(22)** Glutamate 5-kinase (AA347_00930); **(23)** Gamma-glutamyl phosphate reductase (AA347_01121); **(24)** Ornithine aminotransferase (AA347_01752); **(25)** Pyrroline-5-carboxylate reductase (AA347_01948); **(26)** Aspartate aminotransferase (AA347_01647); **(27)** Aspartate ammonia-lyase (AA347_00952); **(28)** L-asparaginase 2 (AA347_00953); **(29)** argininosuccinate synthase (AA347_01336); **(30)** Argininosuccinate lyase (AA347_01731); **(31)** Biosynthetic arginine decarboxylase (AA347_01873); **(32)** agmatinase (AA347_00964); **(33)** spermidine synthetase (AA347_00329); **(34)** aspartate kinase (AA347_01503); **(35)** aspartate-semialdehyde dehydrogenase (AA347_01039); **(36)** homoserine dehydrogenase (AA347_00407); **(37)** homoserine kinase (AA347_00208); **(38)** threonine synthase (AA347_00183); **(39)** Homoserine O-acetyltransferase (AA347_01182); (40) Cystathionine gamma-synthase (AA347_01684).

*Arcobacter thereius* LMG24486^T^ has a limited capacity to catabolize amino acids, as only genes involved in aspartate, L-glutamate, and serine catabolism were found in its genome (Fig 2).

A gene encoding aspartate aminotransferase was found in the genome of *A*. *thereius* LMG24486^T^ (Fig 2, reaction 26, AA347_01647), which enables this strain to produce oxaloacetate from aspartate. Another important aspartate catabolic pathway involves aspartase (Fig 2, reaction 27. *aspA*, AA347_00952), that allows the production of fumarate and ammonia. Aspartase is a central enzyme in the amino acid catabolism in *C*. *jejuni*, and seems to have an important effect on growth in complex media, as a *C*. *jejuni* mutant lacking a functional aspartase fails to utilize aspartate, glutamate, glutamine and proline [66].

Moreover, aspartase may play a role in the formation of fumarate, an alternative electron acceptor during growth, especially in low oxygen conditions. One of the mechanism of oxygen sensing that has been suggested for aspartase involves the regulator of the CmeABC multidrug efflux transporter, CmeR [67]. In *A*. *thereius* LMG24486^T^, the gene encoding for CmeR is not present, although an anaerobic regulatory protein (AA347_00524) and a transcriptional regulator (AA347_00794) are present, which function typically in response to environmental change. Therefore, they may be involved in modulation of gene expression by the presence of oxygen. Other reactions involving aspartate allow the production of homoserine (Fig 2, reactions 34; 35; 36. AA347_01503; AA347_01039; AA347_00407), threonine (Fig 2, reaction 37; 38; 04. AA347_00208; AA347_00183; AA347_00179) and L-cystathionine (Fig 2, reactions 39; 40. AA347_01182; AA347_01684). Through aspartate, *A*. *thereius* LMG24486^T^ is able to produce the biogenic amines agmatine, putrescine and spermidine (Fig 2, reactions 29; 30; 31; 32; 33. AA347_01336; AA347_01731; AA347_01873; AA347_00964; AA347_00329). Their presence in food is related to the activity of decarboxylase by the microorganism and their occurrence can be detect, most of the time, in meat and meat products [68]. Biogenic amines have been detected in other Gram-negative bacteria, but not in *Arcobacter* yet, and this can represent and essential aspect in prevention in food industry.

Glutamate can be metabolized by *A*. *thereius* LMG24486^T^ by a NADP-dependent glutamate dehydrogenase (Fig 2, reaction 13; AA347_00755) and a glutamate synthase (Fig 2, reaction 14; AA347_01959). Further, ornithine can be formed out of glutamate by an amino acid acetyltransferase (Fig 2, reaction 17; AA347_01854), and L-proline can be formed by a glutamate-5-kinase (Fig 2, reaction 22; AA347_00930). Ornithine can be transported through an ornithine/arginine antiporter.

Serine can be deaminated through a serine deaminase (Fig 2, reaction 4 AA347_00179), a pyridoxal 5-phosphate (PLP)-dependent enzyme that converts serine to pyruvate, thereby generating ammonia. Further, serine can be converted in tryptophan or glycine by a tryptophan synthase (AA347_01111, AA347_01680) and a serine hydroxymethyltransferase (AA347_01745).

#### 2.3. Respiration

*Arcobacter thereius* has previously been described as microaerophilic bacteria with an optimal temperature range for growth of 21 to 30°C. Growth at 37°C in air or in anaerobic conditions was not observed [13]. This seems however to contrast with its natural presence in the pig and duck gut. All 9 *A*. *thereius* strains tested were able to grow and were motile at 28°C in aerobic and microaerobic conditions, but growth as well as motility were completely absent at 37°C and 42°C in all atmospheres tested (Table 3). Remarkably, the genome of *A*. *thereius* LMG24486^T^ harbours full clusters of genes encoding for aerobic and microaerobic respiration. Concerning oxygen-dependent electron transport, a NADH:quinone oxidoreductase (Complex I; AA347_00727—AA347_00740) is present together with the aldehyde dehydrogenase complex (AA347_00801), allowing oxidation of NADH by complex I and reduction of NADP^+^ by an aldehyde dehydrogenase.

A cytochrome bc_1_ complex (AA347_00184; AA347_00185; AA347_00186), a cytochrome c oxidase (AA347_00228; AA347_00230; AA347_00231; AA347_00379), and a complete cluster of genes encoding a F_0_/F_1_ ATPase (AA347_00180; AA347_00936—AA347_00942; AA374_01050) are present, enabling production of ATP from ADP or to generate a transmembrane ion gradient. Three genes encoding a cytochrome c peroxidase were found (AA347_00640; AA347_01278; AA347_00869) which may be important in the detoxification of hydrogen peroxide in the periplasm [64].

In *A*. *thereius* LMG 24486^T^, electrons can be fed to the quinone oxidoreductase by the membrane associated FeNi hydrogenase encoded by *hydABCD* (AA347_00601—AA347_00604), a malate oxidoreductase (AA347_00064) and a variety of other primary substrate dehydrogenases, for example glutamate dehydrogenase, isocitrate dehydrogenase or pyruvate dehydrogenase. The groups of genes *hyaABCD* and *hupLS* encoding for another membrane associate FeNi hydrogenase and for an uptake hydrogenase respectively, result as unique gene for *A*. *butzleri* RM4018; although the gene cluster *hypABCDEF* (AA347_00606 –AA347_00615) is present in both of the species.

In contrast to the results of the growth tests in anaerobic condition, both in the present study (Table 3), as well as in previous ones [11–14], the presence of genes encoding fumarate reductase FrdABC (AA347_00741—AA347_00742) together with the additional presence of the *nap* operon (*napDLFHG*; AA347_00782-AA347_00787), encoding for proteins involved in nitrate reduction, provides the possibility for anaerobic growth using fumarate and nitrate as electron acceptors instead of oxygen [64]. The ability of *A*. *thereius* to reduce nitrate has already been reported by Houf et al. [13] Analysis revealed also the presence of the arsenic resistance operon *ars* (*arsCBR;* AA347_01174; AA347_00273; AA347_00274). The system is regulated by the repressor protein ArsR, while ArsB is the determinant of the membrane efflux protein that confers resistance by pumping arsenic from the cell, and ArsC is an arsenate reductase. The *ars* operon has already been described as part of the *E*. *coli* chromosome [69] and on a plasmid in *Staphylococcus aureus* [70]. The oxyanions of arsenic can be used in anaerobic respiration as terminal electron acceptors, and their oxidation can be coupled with oxidation of other organic substrates (pyruvate, acetate) or hydrogen [71], providing energy for active growth and metabolic activity.

### 3. Clinical relevant features within *Arcobacter thereius* LMG24486^T^

#### 3.1. Lipooligosaccharides

Lipopolysaccharide (LPS) and lipooligosaccharide (LOS) are important constituents of the outer membrane of bacteria and they can have a role as virulence factor or in antibiotic resistance mechanism [72]. *Campylobacter jejuni* has been described as only able to express LOS structure and no LPS [73]. The same capacity was retrieved in the genome sequence of *A*. *thereius* LMG24486^T^ where a lipooligosaccharide (LOS) biosynthesis gene cluster (AA347_01016 –AA347_01027) was found. The structure of this gene cluster resembles the organization of *A*. *butzleri* RM4018 and is present and conserved in all *A*. *thereius* strains sequenced in the present study. The genes *waaC* and *waaF* (AA347_01016; AA347_01027) encode a heptosyltransferase I, which is responsible for the linkage between the L-glycero-D-manno-heptose (HEP) and 3-deoxy-D-manno-octulsonic (KDO) and, for a heptosyltransferase II which catalyses the transfer of a second HEP to HEP I [74,75]. It has been shown in *C*. *jejuni* that a mutation in *waaF* increases the susceptibility to different hydrophobic antibiotics, such as novobiocin, and a mutation in *waaC* results in an incomplete LOS [72,76,77]; also their position in the different genomes of *A*. *thereius* stains sequenced in this study, resemble the organization of *C*. *jejuni* [65]. The *kps* genes involved in the capsular production are not present in *A*. *thereius* genomes, as well as, genes encoding for the O-antigen.

#### 3.2. Flagellum

*Arcobacter thereius* possesses a polar flagellum [13] and all genes involved in flagella biosynthesis (*flhA*, *flhB*, *flhF*, *flgL*, *flgC*, *flgE*, *flgB*, *flgG*, *flgK*, *flgH*, *flgI*, *fliI*, *fliR*, *fliK*, *fliE*, *fliN*, *fliG*, *fliF*, *fliS*, *fliD*, *fliQ*, *fliM*, *flaB*) were found in *A*. *thereius* LMG24486^T^, which were organized into three gene clusters (AA347_00097—AA347_00126; AA347_00256—AA347_00267; AA347_00588—AA347_00589). The same gene organization is also found in the other *A*. *thereius* sequenced, suggesting that this gene region is conserved within this species.

*Arcobacter thereius* LMG24486^T^ contains a complete pseudaminic acid biosynthesis pathway (*pseBCFGHI;* AA347_00580 –AA347_00585), which has been reported to constitute a major virulence factor in *C*. *jejuni* [78]. Interestingly, this gene region contained also an acetylase (AA347_00585), as well as a gene with high similarity to *C*. *jejuni pseH* (AA347_00584), which may be involved in flagellar glycosylation. This pathway was only partially present in *A*. *butzleri* RM4018, as both the *pseG* (encoding for an hydrolase with important function during the fourth step of the pseudoaminic acid biosynthesis pathway [78]) and *pseH* genes were missing, suggesting that this post-translationally modification system through O-linked glycosylation is not functional in *A*. *butzleri* [79].

#### 3.3. Chemotaxis

Interaction between bacteria and the environment is a fundamental aspect for the survival and adaptation of the microorganism. In fact, bacteria have a lot of different mechanisms which enable them to respond to environmental changes. *A*. *thereius* LMG22486^T^ harbours 18 methyl-accepting chemotaxis proteins, nine two-component response regulators, and eight sensor histidine kinases. During the comparison, in the genome of *A*. *butzleri* RM4018 36 sensor histidine kinase, 41 response regulators have been found as unique features of *A*. *butzleri* RM4018 [17]. This indicates that *A*. *thereius* might be less reactive to the surrounding changes, although a full set of genes encoding for chemotaxis proteins were present, including CheARDB (AA347_01490 –AA347_01494), CheV (AA347_01730), CheY (AA347_01490). These proteins play a role in signal transmission from the receptor to the flagellum to regulate or change the bacterial movements.

#### 3.4. Antibiotic resistance

An *in silico* search for genes putatively involved in antibiotic resistance revealed the presence of several genes within the *A*. *thereius* LMG24486^T^ genome that could contribute to antibiotic resistance. For instance, a gene cluster encoding a CmeABC efflux pump was found (AA347_00510 –AA347_00512), which is associated with efflux of various antibiotics in *C*. *jejuni* [80,81]. This gene cluster was found in all *A*. *thereius* strains sequenced (S2 Table). The β-lactam resistance mechanisms of *A*. *thereius* LMG24486^T^ are unclear as no genes encoding for β-lactamases were found, which have been reported before in *A*. *butzleri* [17]. However, an *lrgAB* operon was found (AA347_00278 –AA347_00279), described to be involved in penicillin tolerance in *Staphyloccocus aureus* [82]. Evidence was found for the presence of resistance mechanisms towards quinolones, such as a Thr85Ser mutation in the DNA gyrase subunit A. This mutation has been reported to be responsible for resistance of *Arcobacter cibarius* towards ciprofloxacin [83]. The Thr85Ser mutation in GyrA was also present in strains DU22 (AAX29_01876), 440 (AAX25_00506), and 452 (AAX26_01016), whereas a Thr85Ile mutation was found in strain 11743–4 (AAX28_1873). The latter mutation was considered responsible of conferring a high ciprofloxacin resistance in *C*. *coli*, *A*. *butzleri* and *A*. *cryaerophilus* [83,84]. *Arcobacter thereius* LMG24486^T^ possesses no specific mechanisms towards macrolide resistance, such as the presence of a major outer membrane porin (MOMP) or macrolide resistance-associated mutations. The latter include specific point mutations at positions 2074 and 2075 in the 23S rRNA gene, which are involved in erythromycin resistance in *Campylobacter* species [81,85]. However, MOMP was present in strains *A*. *thereius* 213, *A*. *thereius* 216, *A*. *thereius* 440, *A*. *thereius* 11743–4, and *A*. *thereius* DU22 (S4 Table), indicating a potential resistance towards macrolides, though not phenotypical expressed.

Further, evidence for chloramphenicol resistance was found, as a chloramphenicol O-acetyltransferase was present (*cat*; AA347_01813), which was also observed in the *A*. *thereius* strains 440 and DU22.

Several other antibiotic resistant related genes were found in the other *A*. *thereius* strains sequenced, that were not present in the type strain. For example, a gene encoding adenylyltransferase (*aadA25;* AAD) was found in the genomes of *A*. *thereius* strains 213 (AAW29_01543), 216 (AAW30_00055), DU19 (AAX30_01180), and 11743–4 (AAX28_00760), which is involved in resistance towards streptomycin/spectinomycin in *Pasteurella multocida* [86]. Furthermore, a gene encoding an energy-depended membrane-associated protein TetA (AAX28_02020) was found in the genome of *A*. *thereius* 11743–4. This efflux pump, usually present in Gram-negative bacteria, confers resistance to tetracycline by exporting it out of the cell thereby reducing the intracellular concentration [87]. This is the first report of this protein in the family *Campylobacteriaceae*.

The susceptibility results obtained by gradient strip diffusion for the nine *A*. *thereius* strains are shown in Table 3. Of all strains, 80–100% were susceptible to erythromycin, ciprofloxacin, tetracycline and gentamicin. These results resemble those obtained by the genome analysis. In fact none of the strains harbour the point mutation in the 23 rRNA gene causing a specific resistance to erythromycin and, for tetracycline resistance, only the strain *A*. *thereius* 11743–4 carried the TetA protein. Interesting, the ciprofloxacin susceptibility results showed that only *A*. *thereius* 11743–4, carrying the DNA GyraseA point mutation Thr85Ile, is resistant against ciprofloxacin while, *A*. *thereius* DU22, 440 and 452, that carry the Thr85Ser point mutation remain sensitive towards ciprofloxacin. Streptomycin and chloramphenicol show MICs around breakpoint value, while 80–100% of the strains are resistant to spectinomycin. *A*. *thereius* 11743–4 presented with a divergent resistance pattern in comparison to the other strains, pointing towards acquired multiresistance, which has to be further investigated.

#### 3.5. Virulence associated genes

**Fig 3:**
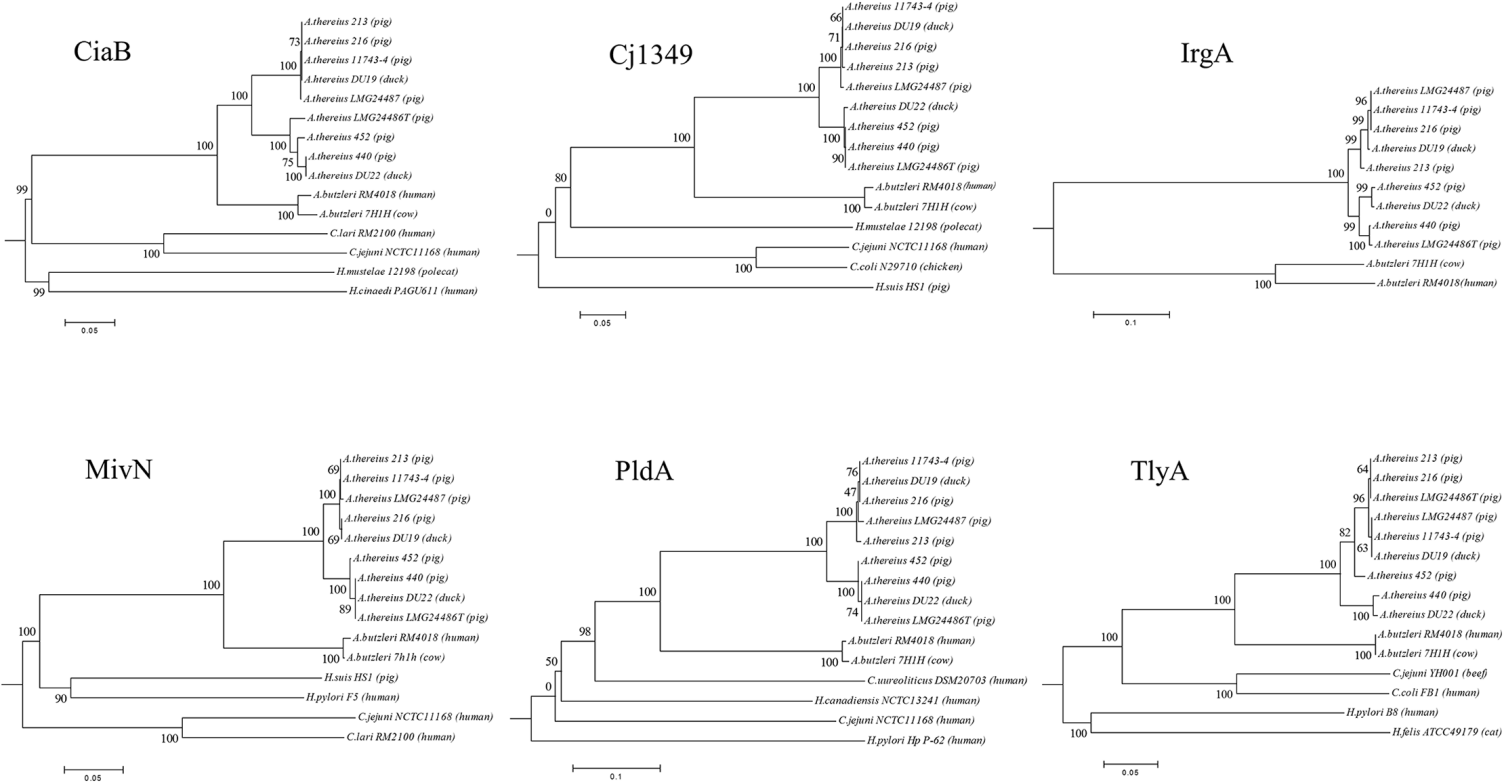
Phylogenetic analysis of the six putative virulence proteins of *Arcobacter thereius*. A Neighbor-Joining phylogenetic tree representing the six putative virulence proteins found in *A*. *thereius*. *Arcobacter*, *Campylobacter* and *Helicobacter* species are included as outgroup. The scale bar represent substitution per site.

Several virulence factors were detected in the genome of *A*. *thereius* LMG24486^T^, among which the fibronectin binding protein Cj1349 (AA347_00304), the invasion protein CiaB (AA347_00973), the virulence factor MviN (AA347_01129), the phospholipase PldA (AA347_01541), the hemolysin TlyA (AA347_01277), and the enterobactin receptor IrgA (AA347_01908 and AA347_01909) [17,88]. The presence of two copies of the latter gene is in contrast with the genome of *A*. *butzleri* RM4018, which encodes a putative siderophore esterase IroE adjacent to a single copy of the IrgA gene [17]. Next to the absence of IroE, other virulence factors reported in *A*. *butzleri*, *A*. *cryaerophilus* and *A*. *skirrowii* such as HecAB [88] and CadF [16,88], were not found in *A*. *thereius* LMG24486^T^. The virulence factors present in the other eight *A*. *thereius* strains sequenced in this study were identical to the type strain (S3 Table), except for *A*. *thereius* DU22, which possessed the *hecAB* virulence genes (AAX29_00055 –AAX29_00056). The putative virulence genes *cj1349*, *ciaB*, *mviN*, *pldA*, *tlyA* and *irgA* were conserved within the species *A*. *thereius* (Fig 3). Although the presence of these virulence factor were reported before in *Arcobacter* species [17,88], this is the first report of their occurrence in *A*. *thereius*. Indeed, traditional PCR detection methods fail when applied on *A*. *thereius* strains [16], which might be related to differences in DNA composition due to evolutionary modifications of the virulence genes.

### 4. Analysis of the genome variability within *A*. *thereius*

**Fig 4:**
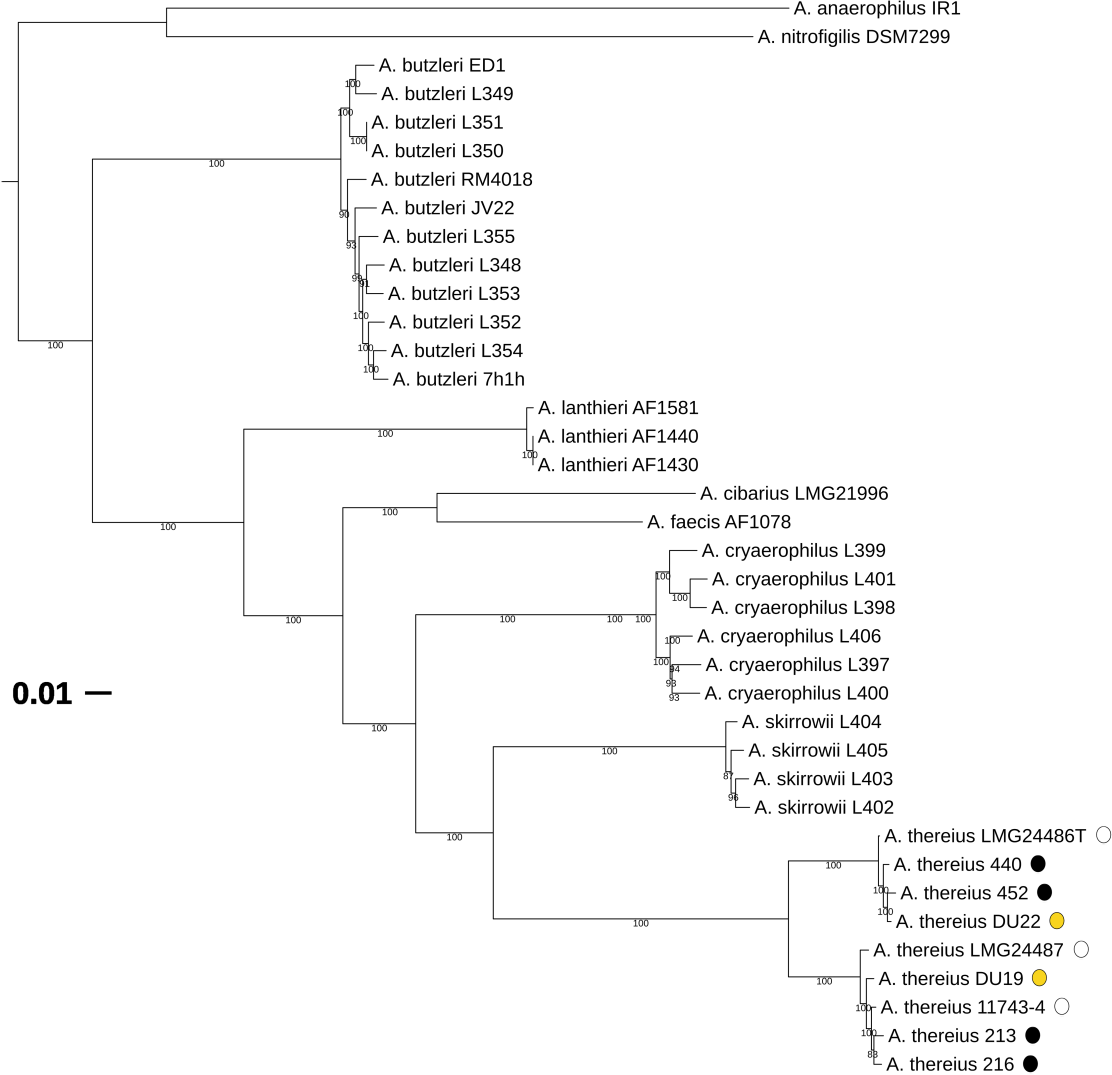
Phylogenetic tree of *Arcobacter genus*. A maximum likelihood phylogenetic tree constructed using 239 single-copy orthologs and representing the *A*. *thereius* strains sequenced in this study and all other *Arcobacter* genomes available. *C*. *jejuni* was not included in the phylogenetic tree.“White dots” = *A*. *thereius* isolated from piglet aborted foetus; “Black dots” = *A*. *thereius* isolated from pig faeces; “Yellow dots” = *A*. *thereius* isolated from cloaca content duck.

Comparison of the genomes from all *A*. *thereius* strains sequenced in the present study, displays a similar genomic architecture, although a few structural rearrangements were found (S4 Table, S1 Fig). We examined whether the genome content of *A*. *thereius* strains isolated from the cloaca of ducks were distinct from those isolated from pig faeces or the tissue of aborted piglets. A phylogenetic tree based on the alignment of 239 single-copy orthologs was constructed (Fig 4). The interspersed positions of the pig faeces, piglet tissue and duck cloaca isolates on the species tree, together with the short length of the branches, indicate that a single, homogenous population of *A*. *thereius* is maintained in the different animal populations. This hypothesis is further supported by the fact that there were only minor differences in the distribution of genes into clusters of orthologous groups of proteins (COG) functional categories across genomes (S2 Fig). This result indicate a limited functional variability among different *A*. *thereius* strains. For example, only two genes without a clear link to disease or habitat were present in both strains isolated from cloaca from ducks but absent in all other strains, encoding a serine hydroxymethyltransferase (DU19: AAX30_00966 and DU22: AAX29_01527) and a UDP-N-acetyl-D-glucosamine 6-dehydrogenase (DU22: AAX29_01729 and DU19: AAX30_01828). The seven strains isolated from pigs contained two genes which were absent in all strains originating from duck cloaca, both encoding hypothetical proteins which are adjacent to each other. The genome of *A*.*thereius* LMG24486^T^ harboured 394 accessory genes (S5 Table), i.e., genes without a homolog in the other *A*. *thereius* genomes. Most of these genes were organised among 18 islands. These strain-specific genes included 26 genes coding for phage integrases, tyrosine recombinases, prophage integrases and CRISPR- associated endonucleases. Further, two complete genes clusters coding for the FeNi hydrogenase and for the HypABCDEF complex (AA347_00599 –AA347_00602; AA347_00604 –AA347_00613; discussed above) were detected in the type strain. The occurrence of two unique additional hydrogenase might enable this strain to obtain energy in microaerobic environments. Because they are not found in related, pathogenic strains, these genes, unique to *A*. *thereius* LMG24486^T^_,_ might confer an advantage in some aspects of *A*. *thereius*’ behaviour, but it is unlikely that they play a role in pathogenicity.

Though in the initial characterization of the species, DNA-DNA hybridization of strains LMG24486^T^ and *A*. *thereius* LMG24487 exhibited a mean DNA-DNA relatedness value of 79% [13], *in silico* DDH as well as Average Nucleotide Identity (ANI) analysis suggest a separation into two species, each inclosing one of the strains. The *in silico* DDH values and the ANI data for the nine *A*. *thereius* strains are shown in Table 5 and Table 6. Combining these findings with the results obtained by the phylogenetic analysis, there is indeed a consistent split into two closely related subclusters within the set of *A*. *thereius* strains examined. However, strains of both clusters have been included in the previous characterization of the species [13], with over 60 phenotypic characteristics determined. No differential diagnostic test (a strict requirement for the description of a new species) could be identified. Furthermore, there is currently also no geographic, biological niche, genome content, nor clinical relevance indicating the need for the relocation into a new species. Because the taxonomic criteria for the creation of a new species are not fulfilled, the strains have to be considered as closely related genotypes of the same species. However, recently, a study based on ANI and *is*DDH data analysis on the strains included in the present study proposed the classification into a new species, *Arcobacter porcinus*, including the majority of strains previously identified as *A*. *thereius* [89]. Further studies seem necessary in order to provide a clear overview in the taxonomy of the *Arcobacter* genus.

**Table 5:** Estimation of *in silico* DDH for the nine *A*. *thereius* strains. The confidential interval is shown in square brackets. *A*. *butzleri* RM4018 and *A*. *cibarius* LMG21996 were included as references. The symbol “*” is added when the same genomes are compared.

	*A*. *thereius* LMG24486^T^	*A*. *thereius* LMG24487	*A*. *thereius* 213	*A*. *thereius* 216	*A*. *thereius* 440	*A*. *thereius* 452	*A*. *thereius* 11743–4	*A*. *thereius* DU22	*A*. *thereius* DU19	*A*. *cibarius* LMG21996	*A*. *butzleri* RM4018
*A*. *thereius* LMG24486^T^	*	51.10 [48.5–53.8%]	51.40 [48.7–54.1%]	51.50 [48.8–54.1%]	91.30 [89.2–93.1%]	88.90 [86.4–90.9%]	50.90 [48.2–53.5%]	90.60 [88.3–92.5%]	52.40 [49.7–55.1%]	22.30 [20.1–24.8%]	20.60 [18.4–23.1%]
*A*. *thereius* LMG24487	51.10 [48.5–53.8%]	*	86.40 [83.7–88.6%]	87.40 [84.9–89.6%]	50.20 [47.5–52.8%]	51.10 [48.4–53.7%]	84.00 [81.3–86.5%]	49.30 [46.7–52%]	89.30 [86.9–91.3%]	22.30 [20–24.7%]	20.70 [18.4–23.1%]
*A*. *thereius* 213	51.40 [48.7–54.1%]	86.40 [83.7–88.6%]	*	92.40 [90.4–94.1%]	50.10 [47.4–52.7%]	49.60 [47–52.2%]	92.50 [90.5–94.1%]	49.30 [46.7–51.9%]	88.70 [86.3–90.8%]	22.00 [19.7–24.5%]	20.60 [18.4–23%]
*A*. *thereius* 216	51.50 [48.8–54.1%]	87.40 [84.9–89.6%]	92.40 [90.4–94.1%]	*	49.90 [47.3–52.5%]	49.90 [47.3–52.5%]	91.50 [89.4–93.3%]	49.60 [47–52.3%]	91.10 [88.9–92.9%]	22.10 [19.8–24.5%]	20.50 [18.3–22.9%]
*A*. *thereius* 440	91.30 [89.2–93.1%]	50.20 [47.5–52.8%]	50.10 [47.4–52.7%]	49.90 [47.3–52.5%]	*	88.60 [86.1–90.7%]	49.80 [47.2–52.4%]	90.50 [88.3–92.4%]	50.90 [48.3–53.6%]	22.10 [19.8–24.5%]	20.40 [18.2–22.8%]
*A*. *thereius* 452	88.90 [86.4–90.9%]	51.10 [48.4–53.7%]	49.60 [47–52.2%]	49.90 [47.3–52.5%]	88.60 [86.1–90.7%]	*	49.90 [47.3–52.5%]	90.10 [87.8–92%]	50.20 [47.6–52.9%]	22.30 [20–24.7%]	20.60 [18.4–23%]
*A*. *thereius* 11743–4	50.90 [48.2–53.5%]	84.00 [81.3–86.5%]	92.50 [90.5–94.1%]	91.50 [89.4–93.3%]	49.80 [47.2–52.4%]	49.90 [47.3–52.5%]	*	49.50 [46.9–52.2%]	90.70 [88.5–92.5%]	22.20 [20–24.7%]	20.60 [18.4–23%]
*A*. *thereius* DU22	90.60 [88.3–92.5%]	49.30 [46.7–52%]	49.30 [46.7–51.9%]	49.60 [47–52.3%]	90.50 [88.3–92.4%]	90.10 [87.8–92%]	49.50 [46.9–52.2%]	*	50.20 [47.6–52.9%]	22.10 [19.9–24.6%]	20.50 [18.3–23%]
*A*. *thereius* DU19	52.40 [49.7–55.1%]	89.30 [86.9–91.3%]	88.70 [86.3–90.8%]	91.10 [88.9–92.9%]	50.90 [48.3–53.6%]	50.20 [47.6–52.9%]	90.70 [88.5–92.5%]	50.20 [47.6–52.9%]	*	22.10 [19.9–24.6%]	20.60 [18.4–23%]
*A*. *cibarius* LMG21996	22.30 [20.1–24.8%]	22.30 [20–24.7%]	22.00 [19.7–24.5%]	22.10 [19.8–24.5%]	22.10 [19.8–24.5%]	22.30 [20–24.7%]	22.20 [20–24.7%]	22.10 [19.9–24.6%]	22.10 [19.9–24.6%]	*	22.00 [19.7–24.4%]
*A*. *butzleri* RM4018	20.60 [18.4–23.1%]	20.70 [18.4–23.1%]	20.60 [18.4–23%]	20.50 [18.3–22.9%]	20.40 [18.2–22.8%]	20.60 [18.4–23%]	20.60 [18.4–23%]	20.50 [18.3–23%]	20.60 [18.4–23%]	22.00 [19.7–24.4%]	*

**Table 6:** Estimation of the Average Nucleotide Identity (ANI) for the nine *A*. *thereius* strains with *A*. *butzleri* RM4018 and *A*. *cibarius* LMG21996 were included as outgroup. ANI results are based on BLAST+ analysis and expressed as [aligned nucleotides] [%].The symbol “*” is added when the same genomes are compared.

	*A*. *thereius* 213	*A*. *thereius* 216	*A*. *thereius* 440	*A*. *thereius* 452	*A*. *thereius* 11743–4	*A*. *thereius* LMG24486^T^	*A*. *thereius* LMG24487	*A*. *thereius* DU19	*A*. *thereius* DU22	*A*. *cibarius* LMG21996	*A*. *butzleri* RM4018
*A*. *thereius* 213	*	99.00 [82.37]	92.86 [79.75]	92.70 [78.21]	99.00 [81.73]	93.23 [78.66]	98.42 [81.02]	98.66 [81.84]	92.66 [78.93]	79.29 [58.23]	78.09 [58.57]
*A*. *thereius* 216	99.01 [83.13]	*	92.96 [78.20]	92.95 [77.88]	98.93 [83.68]	93.28 [79.40]	98.46 [81.31]	98.86 [82.15]	92.80 [77.83]	79.31 [59.50]	78.07 [58.59]
*A*. *thereius* 440	92.70 [73.25]	92.63 [71.74]	*	98.61 [77.38]	92.61 [73.46]	98.82 [79.48]	92.75 [74.52]	93.00 [74.12]	98.70 [77.47]	79.20 [52.95]	77.94 [53.52]
*A*. *thereius* 452	92.59 [70.15]	92.68 [69.65]	98.54 [75.97]	*	92.65 [70.91]	98.52 [75.45]	93.13 [75.13]	92.90 [70.09]	98.68 [79.29]	79.19 [51.52]	77.98 [52.79]
*A*. *thereius* 11743–4	98.93 [74.97]	98.79 [76.45]	92.68 [73.48]	92.83 [71.99]	*	93.01 [73.86]	97.92 [77.46]	98.58 [76.17]	92.56 [72.24]	79.33 [53.99]	77.93 [53.60]
*A*. *thereius* LMG24486^T^	93.13 [74.00]	93.03 [74.50]	98.83 [81.40]	98.64 [78.40]	93.00 [75.70]	*	93.09 [76.33]	93.42 [74.88]	98.70 [81.43]	79.35 [54.60]	78.07 [55.75]
*A*. *thereius* LMG24487	98.24 [67.94]	98.16 [68.01]	92.74 [68.30]	93.27 [70.02]	97.76 [71.10]	93.05 [68.38]	*	98.40 [70.65]	92.70 [67.93]	79.28 [49.09]	78.03 [49.03]
*A*. *thereius* DU19	98.46 [79.05]	98.64 [78.22]	93.16 [77.35]	93.06 [74.80]	98.56 [79.66]	93.48 [76.32]	98.57 [80.70]	*	92.99 [75.14]	79.41 [55.69]	77.98 [55.78]
*A*. *thereius* DU22	92.57 [69.46]	92.51 [68.54]	98.79 [74.82]	98.80 [77.81]	92.58 [69.64]	98.70 [76.84]	92.60 [71.95]	92.82 [69.22]	*	79.30 [50.72]	77.90 [52.40]
*A*. *cibarius* LMG21996	79.18 [49.03]	79.19 [49.63]	79.24 [49.10]	79.13 [48.70]	79.25 [49.71]	79.21 [49.25]	79.35 [49.72]	79.24 [49.63]	79.20 [48.75]	*	79.10 [54.71]
*A*. *butzleri* RM4018	78.18 [45.41]	78.17 [45.24]	78.06 [46.23]	78.13 [46.12]	78.20 [45.29]	78.08 [46.42]	78.03 [45.87]	78.11 [45.50]	78.08 [46.43]	79.13 [50.47]	*

MLST analysis on the nine *A*. *thereius* genomes revealed the consistent presence of the loci *aspA*, *atpA*, *glnA*, *gltA*, *pmg* and *tkt*. In contrast *glyA* locus was lacking in *A*. *thereius* 213, 216, 440, 452 and 11743–4, while two copies were present in the genomes of *A*. *thereius* DU19, DU22, LMG24487 and LMG24486^T^ (S3 Fig). The MLST analysis of the concatenated genes confirmed again the presence of two closely related genotypes, as also found by DDH and ANI analysis.

**Fig 5:**
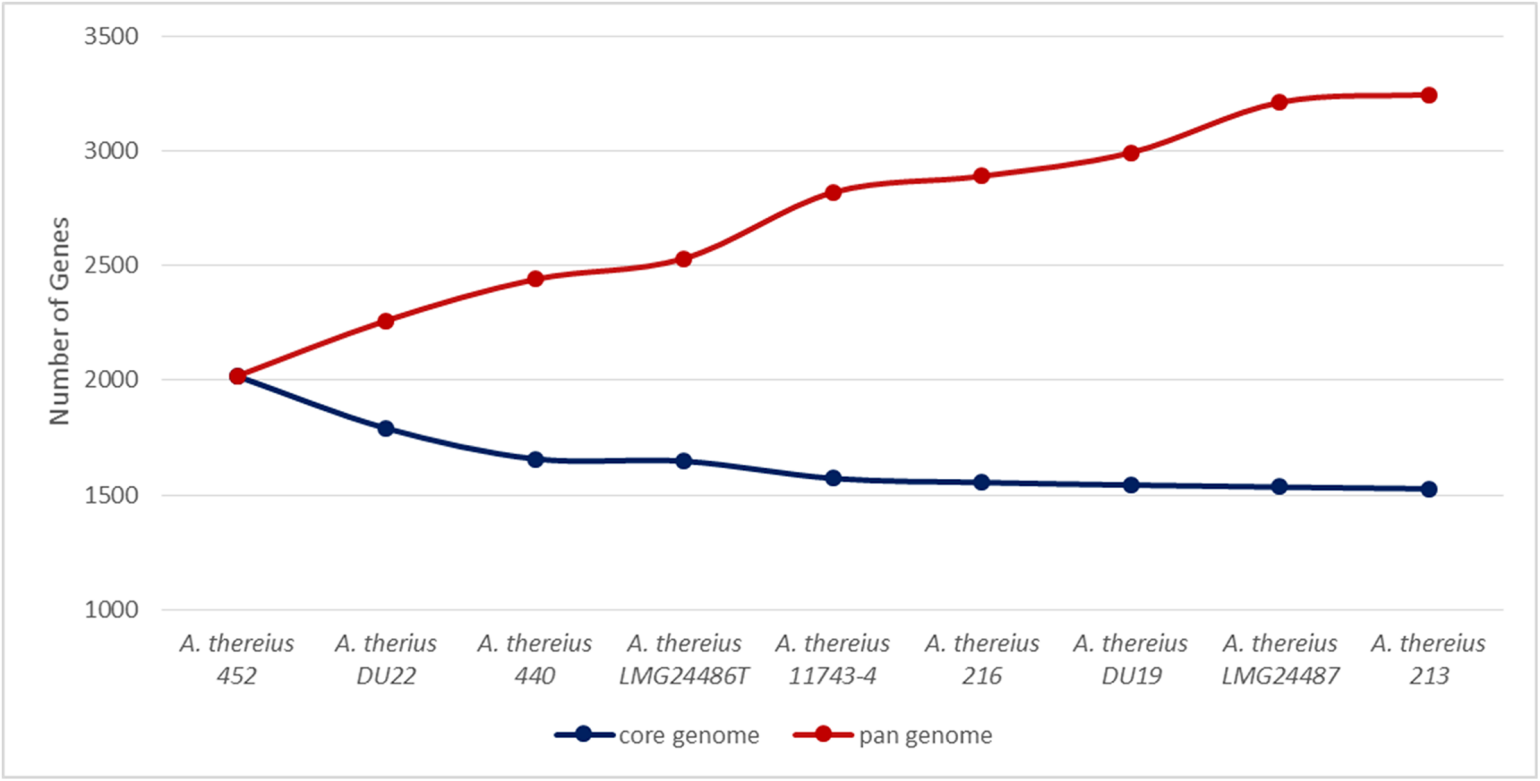
Core and pan genome development plot. Prediction of the development of the core and pan genome for the species *Arcobacter thereius*, based on the nine genome sequences determined in this study. Starting with the first genome, a sequence of core and pan genome sizes is calculated by iteratively adding one genome at time to the comparison and observed value are depicted in full circle [51].

Fig 5 shows a plot of the core and the pan genome of the nine *A*. *thereius* strains included in the present study. Based on EDGAR analysis using one complete genome (*A*. *thereius* LMG24486^T^) and eight draft genome sequences, the *A*. *thereius* core genome showed to have a stable size with the largest proportion of the genes part of the core-genome, while the pan-genome is predicted to be open.

Of the eight *A*. *thereius* genomes sequenced, *A*. *thereius* 440 recovered from pig faeces and LMG24487 isolated from piglet aborted foetus, carried the gene encoding for the zonula occludens toxin (ZOT) (AAX25_01078; AAX27_00411). The ZOT is known to work on the intracellular tight junction and allows pathogenic bacteria, like *Vibrio cholera* or *Neisseria meningitidis*, to increase tissue permeability. Recently, this gene has been detected also in the genome of *Campylobacter concisus* 13826 [90], a non-*jejuni Campylobacter* found in samples of patients with gastrointestinal disorders and now suggested as a potential pathogen [91]. According to Kaakoush et al. [90], the presence of the ZOT gene can have an important role in the pathogenesis of *C*. *concisus*, allowing the bacteria to attach and invade host cells through a paracellular mechanism. It was not detect in other *Arcobacter* species, and further research is needed, taking into account current genome knowledge, to elucidate which genes participate in cell adhesion and invasion.

A gene cluster, coding for type IV secretion system (*virB4*, *virB6*, *virB9*, *virB10*, *virB11*; AAW29_01609; AAW29_01614; AAW29_01615; AAW29_01616; AAW29_001618) was present in the genome of *A*. *thereius* 213 as a singleton. The type IV secretion contributes in a lot of transport like, exchange of genetic material between bacteria, movement of plasmid and injection of virulence factor in the host cell. The subgroup VirB is specific for the T-DNA transfer and is built with 11 different proteins (VirB1-VirB11), but a conserved core of five proteins is always present (VirB4; VirB7; VirB9; VirB10; VirB11) [92]. This secretion system has been described as part of the large plasmid (AC1119) found in *A*. *butzleri* and it could play an important role in gene transfer within *Arcobacter* [60].

### 5. Genome comparison of *A*. *thereius* LMG24486^T^ and *A*. *butzleri* RM4018

**Fig 6:**
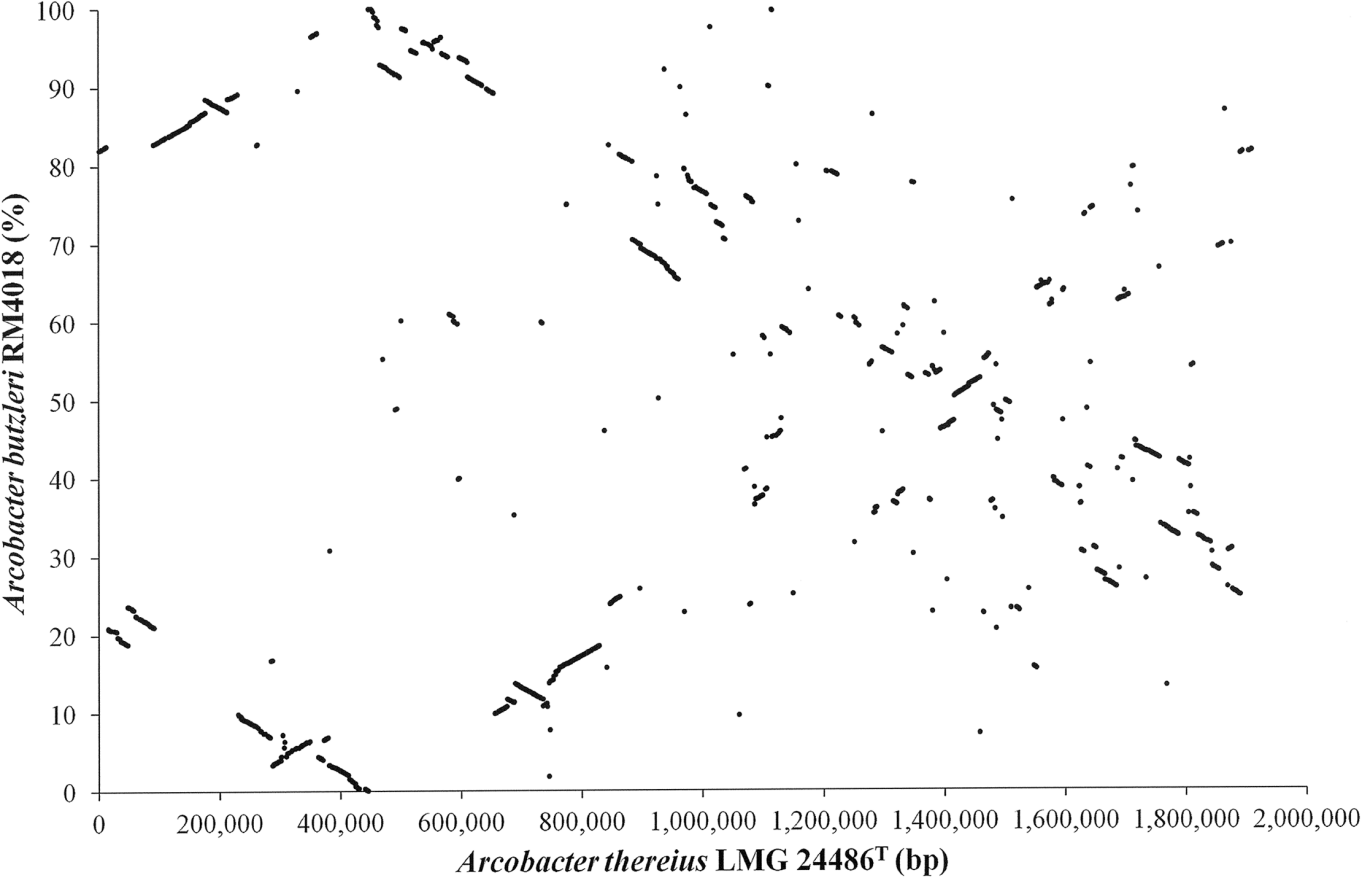
Comparative genome analysis of *Arcobacter* species. Synteny plot depicting the finished genome sequences of *A*. *thereius* LMG24486^T^ and *A*. *butzleri* RM4018. Each dot represents a reciprocal best BLAST hit. For the x-axis, the genomic position is depicted, for the y-axis, 100% corresponds with 2,341,251 bp.

*Arcobacter butzleri* is the most representative human related *Arcobacter* and, after the announcement of its genome, its role as potential human pathogen has been confirmed in several reported cases. The genomes of *A*. *thereius* LMG24486^T^ and *A*. *butzleri* RM4018 show little synteny (Fig 6), which supports the view that *A*. *thereius* appeared to have unique characteristics, different from those previously reported for *A*. *butzleri* [17]. Indeed, the genome sequences of *A*. *thereius* LMG24486^T^ and *A*. *butzleri* RM4018 shared 1474 (ortholog) genes, *A*. *thereius* LMG24486^T^ contained 393 singletons (among which 219 encode hypothetical proteins), and *A*. *butzleri* RM4018 contained 688 singletons (among which 344 encode hypothetical proteins). Besides the differences and similarities already mentioned above, among the singletons of *A*. *butzleri* RM4018 a complete cluster of genes encoding for sulphur uptake and assimilation was present [17]. However, as the *sox* gene cluster, necessary for the sulphur oxidation, were also present in *A*. *thereius* LMG24486^T^ (*soxA*,*Z*,*Y*; AA347_00874—AA347_00876), this strain could be able to oxidise sulphite to sulphate.

Further, a thiamine biosynthetic gene cluster was found (*thiDEHGFS*, AA347_00322 –AA347_00327), enabling thiamine autotrophy in *A*. *thereius* LMG24486^T^. Production of thiamine is an important mechanism because it is an essential cofactor of different metabolic enzymes and it was not present in other *Arcobacter* [17,93] suggesting that this feature is unique for *A*. *thereius*. However, it remains to be determined if this is going to be maintained when genomes of other *Arcobacter* species will be available.

Another interesting difference present as a singleton in *A*. *thereius* LMG24486^T^genome are the type I, II and III restriction endonuclease. These enzymes protect bacteria against invasion of foreign DNA, and differ in their way of recognition and cleavage [94]. Type I restriction enzymes consist in three subunit, HsdS (AA347_01253), HsdM (AA347_0152) and HsdR (AA347_01255; AA347_00856; AA347_00561) and, they are responsible for modification, restriction and sequence recognition [94]. For the type III restriction enzyme only the subunit Res (AA347_01092) has been found in the genome of *A*. *thereius* LMG24486^T^ meaning that this enzyme is not working, although two type II enzymes were harboured (AA347_1323; AA347_01567).

In contrast to *A*. *butzleri* RM4018, the genome sequence of LMG24486^T^ contains the genes of the ectoine biosynthesis pathway (*ectABC*, AA347_00353 –AA347_00355). Ectoine is a compatible solute, important for its function as osmoprotector; it helps microorganism to survive extreme osmotic stress and temperature stress. As *A*. *thereius* LMG 24486^T^ contains also an aspartate kinase (AA347_01497), and an aspartate semialdehyde dehydrogenase (AA347_01037), ectoine may be produced out of aspartate. Further, all six genes involved in urea degradation (*ureABCDEFD*) in *A*. *butzleri* RM4018 [17] were not present in either of the *A*. *thereius* strains.

## Conclusion

Whole genome analysis of nine *A*. *thereius* strains, including the type strain, confirmed that they do not ferment nor oxidize carbohydrates, and energy provision depends rather on a limited group of amino acids. The species is regarded as difficult to grow under laboratory conditions, and growth temperature and atmosphere requirements are not exactly similar to those previous described for *A*. *butzleri*. However, we could not identify the possible genetic determinants for these differences in the genomes of *A*. *thereius*.

*Arcobacter thereius* is predominantly present in the pig intestinal tract, but, due to the apparent lack of virulence factors previously based on specific PCR detection, has not been considered an important species from the food safety perspective so far. However, the species was recently isolated from the stool of human enteritis patients, and the present study reveals the presence of six out of eight virulence associated genes previously reported in *A*. *butzleri*. Further research will elucidate the role of these genes in the potential pathogenicity of *A*. *thereius*.

Comparative genome and phylogenetic analysis of the nine *A*. *thereius* strains revealed the delineation of two closely related subgroups, for which, besides the group including the *A*. *thereius* reference strain, a new species, *A*. *porcinus* has recently be proposed [89]. However, at present, no clear phenotypic or genomic differences are yet identified to support this creation of a new species. Comparative genome analysis with the other human associated species, *A*. *butzleri*, reveals a large correlation, though also unique features are present. The occurrence of virulence associated genes, genes for antibiotic resistance as well as other pathogenic related relevant features, justifies a further exploration of the reservoir, transmission routes, consolidated in a risk assessment in both human and veterinary medicine.

## Supporting information

S1 Fig Alignment with ProgressiveMauve (default parameters) of the nine *A*. *thereius* strains sequenced. (TIF)

S2 Fig Distribution of genes in COG functional categories. The number of genes in each categories was compared among the nine *A*. *thereius* strains sequenced. No significant differences in the distribution of genes belonging to a functional category among the different strains has been found (see Materials and Method). (C) = Energy production and conversion; (D) = Cell cycle control, cell division, chromosome partitioning; (E) = Amino acid transport and metabolism; (F) = Nucleotide transport and metabolism; (G) = Carbohydrate transport and metabolism; (H) = Coenzyme transport and metabolism; (I) = Lipid transport and metabolism; (J) = Translation, ribosomal structure and biogenesis; (K) = Transcription; (L) = Replication, recombination and repair; (M) = Cell/wall/membrane/envelope biogenesis; (N) = Cell motility; (O) = Posttranslational modification, protein turnover, chaperones; (P) = Inorganic ion transport and metabolism; (Q) = Secondary metabolites biosynthesis, transport and catabolism; (R) = General function protection only; (S) = Function unknown; (T) = Signal transduction mechanisms; (U) = Intracellular trafficking, secretion, and vascular transport; (V) = Defence mechanisms.
(PDF)

S3 Fig Phylogenetic analysis of the six genes used in the MLST typing scheme for *Arcobacter* spp. A neighbour-joining phylogenetic tree representing the six genes involved in the MLST method. *Arcobacter* and *Campylobacter* species are included as outgroup. For *Campylobacter jejuni* ATCC43439 the gene *uncA* (*atpA*) has been used.
(TIF)

S1 Table CRISPR sequence of *A*. *thereius* LMG24486^T^ genome. (XLSX)

S2 Table Position of the genes involved in the antibiotic resistance in the different *A*. *thereius* strains. (XLSX)

S3 Table Position of the virulence genes in the different *A*. *thereius* strains. (XLSX)

S4 Table General features of the nine *A*. *thereius* strains sequenced in this study. (XLSX)

S5 Table Accessory genes of *A*. *thereius* LMG24486^T^ organise within 18 clusters. (XLSX)
