# Supplementary figures and images for: Characterization of the emerging zoonotic pathogen Arcobacter thereius by whole genome sequencing and comparative genomics
Source: PLoS One. 2017 Jul 3;12(7):e0180493. doi: 10.1371/journal.pone.0180493 (PMC5495459; doi:10.1371/journal.pone.0180493)

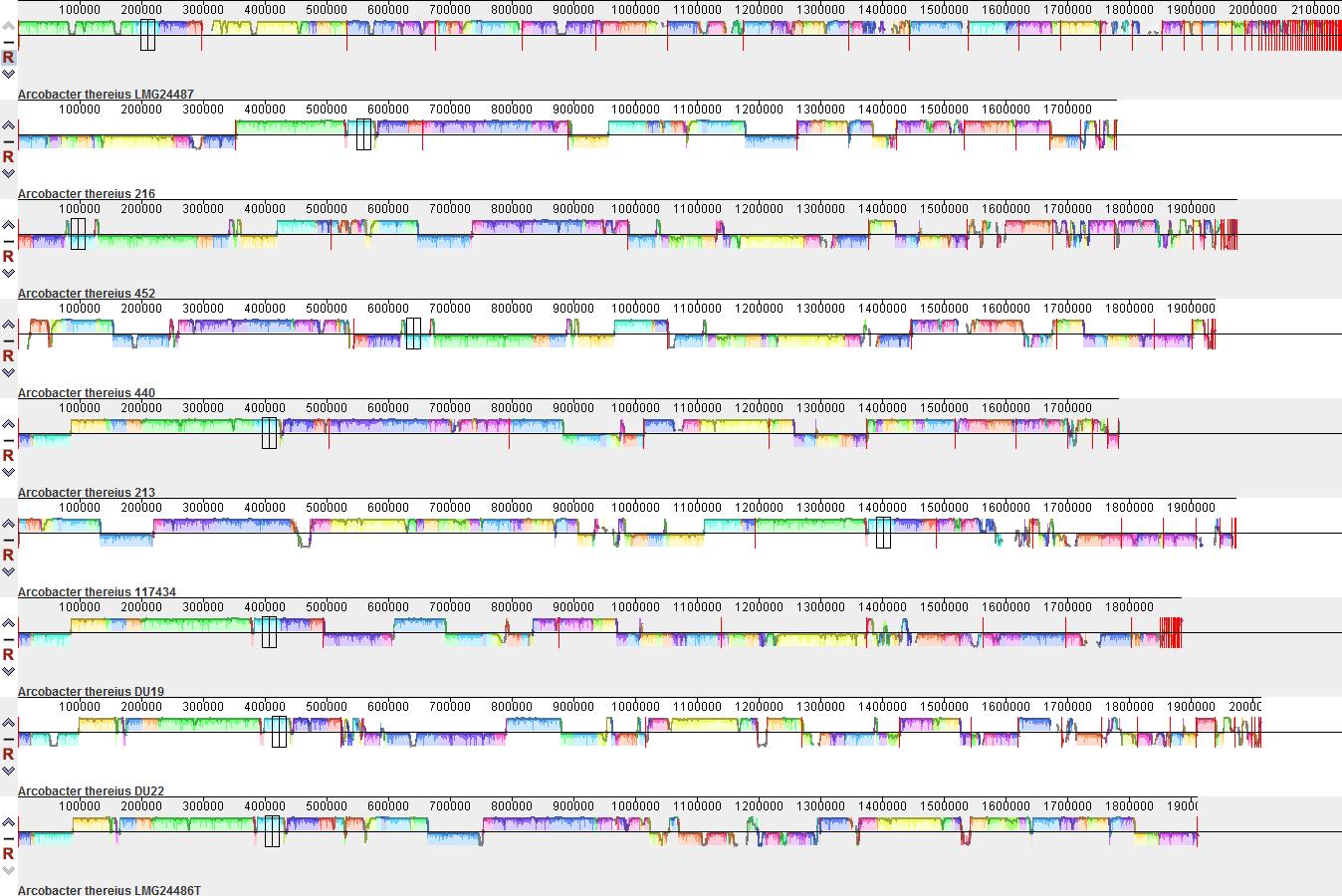

Supplement: S1 Fig — (TIF) [file pone.0180493.s001.tif]

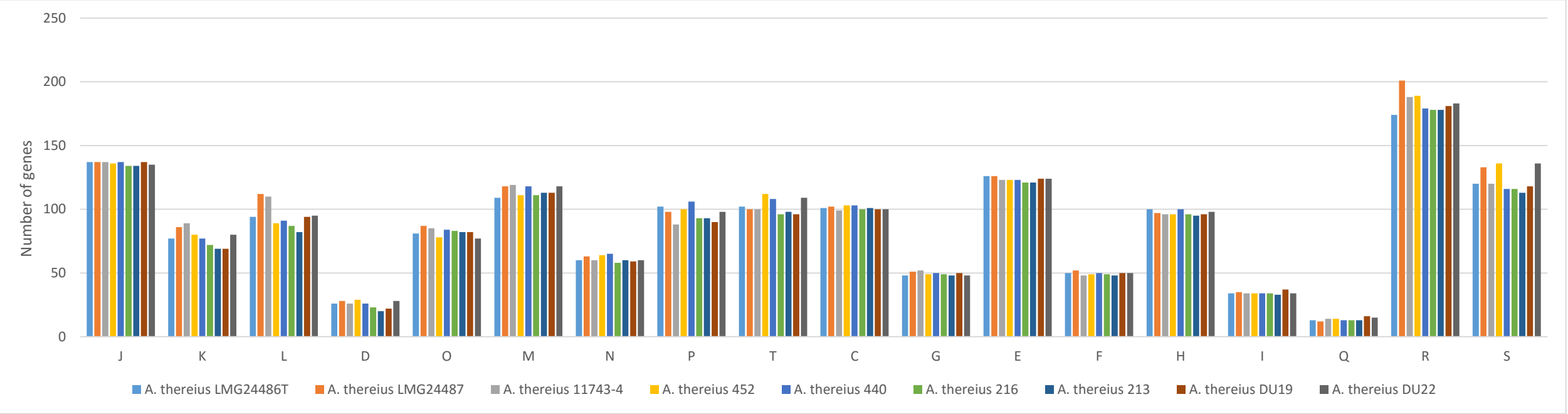

Supplement: S2 Fig — The number of genes in each categories was compared among the nine A. thereius strains sequenced. No significant differences in the distribution of genes belonging to a functional category among the different strains has been found (see Materials and Method). (C) = Energy production and conversion; (D) = Cell cycle control, cell division, chromosome partitioning; (E) = Amino acid transport and metabolism; (F) = Nucleotide transport and metabolism; (G) = Carbohydrate transport and metabolism; (H) = Coenzyme transport and metabolism; (I) = Lipid transport and metabolism; (J) = Translation, ribosomal structure and biogenesis; (K) = Transcription; (L) = Replication, recombination and repair; (M) = Cell/wall/membrane/envelope biogenesis; (N) = Cell motility; (O) = Posttranslational modification, protein turnover, chaperones; (P) = Inorganic ion transport and metabolism; (Q) = Secondary metabolites biosynthesis, transport and catabolism; (R) = General function protection only; (S) = Function unknown; (T) = Signal transduction mechanisms; (U) = Intracellular trafficking, secretion, and vascular transport; (V) = Defence mechanisms. (PDF) [file pone.0180493.s002.pdf]

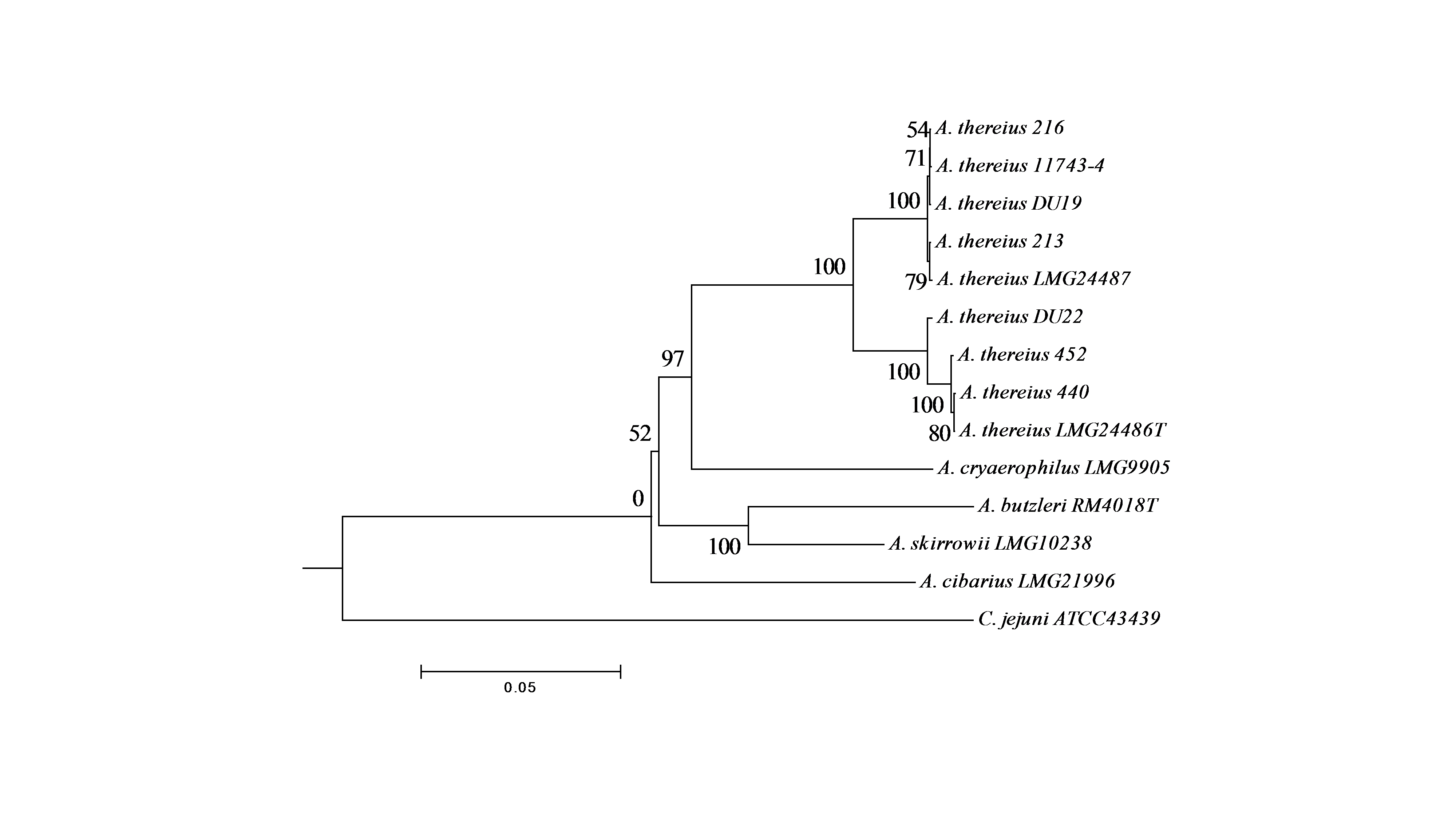

Supplement: S3 Fig — A neighbour-joining phylogenetic tree representing the six genes involved in the MLST method. Arcobacter and Campylobacter species are included as outgroup. For Campylobacter jejuni ATCC43439 the gene uncA (atpA) has been used. (TIF) [file pone.0180493.s003.tif]
